# Novel Poly(ester urethane urea)/Polydioxanone Blends: Electrospun Fibrous Meshes and Films

**DOI:** 10.3390/molecules26133847

**Published:** 2021-06-24

**Authors:** Kiran R. Adhikari, Inessa Stanishevskaya, Pablo C. Caracciolo, Gustavo A. Abraham, Vinoy Thomas

**Affiliations:** 1 Department of Physics, University of Alabama at Birmingham, Birmingham, AL 35294, USA; kiranraj@uab.edu; 2 Center for Nanoscale Materials and Biointegration (CNMB), University of Alabama at Birmingham, Birmingham, AL 35294, USA; 3 Rensselaer Polytechnic Institute, Troy, NY 12180, USA; Inessa.stanish@gmail.com; 4 Instituto de Investigaciones en Ciencia y Tecnología de Materiales, INTEMA (UNMdP-CONICET), Av. Juan B. Justo 4302, B7608FDQ Mar del Plata, Argentina; pcaracciolo@fi.mdp.edu.ar (P.C.C.); gabraham@fi.mdp.edu.ar (G.A.A.); 5 Department of Materials Science and Engineering, University of Alabama at Birmingham, Birmingham, AL 35294, USA

**Keywords:** polyurethane urea, nanostructure, electrospinning

## Abstract

In this work, we report the electrospinning and mechano-morphological characterizations of scaffolds based on blends of a novel poly(ester urethane urea) (PHH) and poly(dioxanone) (PDO). At the optimized electrospinning conditions, PHH, PDO and blend PHH/PDO in Hexafluroisopropanol (HFIP) solution yielded bead-free non-woven random nanofibers with high porosity and diameter in the range of hundreds of nanometers. The structural, morphological, and biomechanical properties were investigated using Differential Scanning Calorimetry, Scanning Electron Microscopy, Atomic Force Microscopy, and tensile tests. The blended scaffold showed an elastic modulus (~5 MPa) with a combination of the ultimate tensile strength (2 ± 0.5 MPa), and maximum elongation (150% ± 44%) in hydrated conditions, which are comparable to the materials currently being used for soft tissue applications such as skin, native arteries, and cardiac muscles applications. This demonstrates the feasibility of an electrospun PHH/PDO blend for cardiac patches or vascular graft applications that mimic the nanoscale structure and mechanical properties of native tissue.

## 1. Introductions

Tissue regeneration, replacement, or repair of the damaged tissues, organs, or body structures is the major goal of tissue engineering [1]. With the scaffold being a major component of tissue engineering, mimicking closely the morphological, mechanical, and functional properties of native extracellular matrices (ECM) has attracted a great deal of attention in the fabrication of scaffolds for tissue regeneration [2,3,4,5,6,7,8,9,10,11,12]. Electrospinning is an enabling nanofabrication technique for creating nanofibrous tissue scaffolds having an extracellular matrix-like environment with tailored properties for tissue engineering and drug delivery applications [6,13,14,15,16,17,18,19]. The process of electrospinning has previously been described in detail [20,21], however, in short, it is a process of drawing fibers from viscoelastic fluid in which an electromagnetic field created by a high-voltage source causes the charged viscoelastic polymer solution-drop to jet towards a grounded surface and deposit as a non-woven fibrous mesh after undergoing a whipping instability motion. Fibers with diameters in the range from several micrometers down to less than 100 nm can be produced, and some studies reported the diameter of electrospun fibers to be as low as 10 nm [22,23]. Collagens, a major protein component of ECM found in both soft and hard tissues, are in the form of fibers with diameters in the range of 50 to 500 nm [24]. Therefore, nanofibers are only synthetic analogs to native ECM and cells whose behavior are more similar physiologically on these biomimetic meshes [25]. Moreover, the manufacturing control over the diameter and pore size of these mesh structures in electrospun fibers and meshes provides an extra degree of flexibility for tissue engineering applications. This control over the fibrous structures and their diameter can be achieved by a myriad of control mechanisms over the experimental parameters and a range of material choices during the electrospinning process [26].

The choice of scaffolding materials is as important as the fabrication process. Among the various materials employed for tissue engineering scaffolds, synthetic biodegradable polyesters such as polycaprolactone (PCL), polyglycolide (PGA), polylactide (PLA), and their copolymers have drawn considerable attention due to their processibility and proven use as absorbable suture materials [27,28,29]. These classical aliphatic polyesters and their copolymers are relatively stiff and may not be very well-suited for the engineering of soft and flexible tissues. Moreover, in vivo study of pure PCL-based small-diameter vascular grafts in rat systemic circulation revealed that foci of chondroid metaplasia were located in the neointima of all implanted grafts after 6 weeks [30]. This study suggested the local hypoxia accompanied by the acidic degradation products is suspected to be the stimulus for overexpression of transforming growth factor-beta 1 (TGF-β1) from the smooth muscle cells (SMCs) and/or endothelium to induce chondroid metaplasia and these metaplastic areas were found to be calcified after 24 weeks.

The scaffolds intended for use in cardiac and vascular tissue regeneration must undergo repeated cyclic flexing in accordance with the systole and diastole phases of the cardiac cycle. Scaffolds fabricated from elastomeric materials can withstand the action of repeated stress and load and undergo an elastic recovery with little or no hysteresis. Segmented poly(urethane)s and poly(ester urethane urea)s (PEUU) are elastomeric polymers with favorable biocompatibility, soft-tissue mechanical properties, controlled degradation characteristics, and have extensive diverse structure and properties [31,32]. Degradation rates of PEUU can be accelerated to levels relevant for tissue engineering applications by introducing hydrolytically labile components into either the hard or soft segments of the polymer backbone [33,34]. In previous studies, Caracciolo et al. [35,36] have reported the preparation, characterization of thermal and mechanical properties, and in vitro biological properties of aliphatic segmented poly(ester urethane urea) (PHH) based on poly(ε-caprolactone) (PCL), hexamethylene diisocyanate (HDI), and novel aliphatic chain extenders containing urea functional groups. The advantage of using this material in bio-applications is well-documented in the literature. The tissue compatibility of these materials is due to their excellent protein adsorption and cellular interaction at the interface [35,37]. In this work, PHH was blended with polydioxanone (PDO) and electrospun into fibrous scaffolds.

PDO is a semi-crystalline, biodegradable polymer commonly used as bioabsorbable suture material in clinical use, whose mechanical strength is capable of withstanding pulsatile blood flow. It also possesses shape memory, which is a very attractive material for vascular graft applications due to its various advantageous characteristics like its ease of handling, ease of suture placement, suture retention, and biocompatibility [38,39,40]. Moreover, the presence of an ether bond and an additional methylene group in its molecular structure gives polydioxanone more flexibility than PGA and PLA. Polydioxanone and its copolymer materials (MonoFlex) have shown a reduction of ~50% of strength between 4 and 6 weeks in vivo and is absorbed in about 6 months with minimal inflammatory reactions with lower absorption rates than sutures composed of PGA and poly(glycolic-co-lactic acid) (PGLA) [41]. Previously, electrospun hybrid vascular scaffolds of blends of PDO with vascular proteins such as gelatin and/or elastin [38,39,42] have been studied. Recently, the application of PDO has been extensively studied by various researchers. Goonoo et al. reported the fabrication and drug delivery applications of PDO-based electrospun mats. [43] They reported the application of the shape memory property of PDO materials for the fabrication of ECM mimicking mat structures. In another study, Song et al. reported the aligned electrospun fibers mats fabricated with PDO as core material and laminin as shell materials and reported their application in neurogenesis [44]. In this study, we electrospun and compared the properties of fibrous scaffolds and the corresponding non-porous film counterparts of PHH/PDO blends. Using the blends of these materials will enable us to control the mechano-physical properties as well as the degradation behaviors. The electrospun fibrous mats were prepared in this study keeping a wide variety of applications in mind. The non-aligned fibrous structure in the mats is reported to be applicable in applications like vascular grafts, skin implants, etc., whereas the aligned fiber in the mat is reported to be applicable in neural tissue regeneration or applications where aligned tissue structure is warranted [45,46,47,48,49]. The application of elastomeric polymers for cardiac patch application has been widely reported over the year. Mousa et al., in their study, reported the fabrication and three-layered nanofibrous patch and reported the patches with adequate mechanical properties and showed the biocompatibility [50]. Very recently, Beck et al. has shown the PCL- and PU-based blend to fabricate the cardiac patch and has shown excellent cell infiltration [51]. In our study, e-scaffolds were characterized for their biomechanical properties in both dry and hydrated conditions and structural and morphological properties for soft-tissue engineering applications so that the electrospun fiber and mesh structure mimic the properties of the native tissue structures. In this paper, we compare the mechanical behavior, porosity, and PBS uptake of the electrospun mat to their non-porous counterpart films, as electrospun mats have been shown to have ECM-mimicking structure, which is very vital in the biomedical application.

## 2. Materials and Methods

### 2.1. Polyurethane Synthesis

The PHH used for this study was synthesized from PCL, hexamethylene diisocyanate (HDI), and an aliphatic diurea-diol chain extender (AE-H-AE) based on 2-aminoethanol (AE), according to previously reported procedures [35,36]. The nomenclature for the poly(ester urethane urea) for this study is written as PHH as adopted from these studies based on the chain extender used for modifying the poly(ester urethane urea).

### 2.2. Scaffolds and Films Preparation

Electrospun scaffolds were fabricated by the process of electrospinning solutions using PHH, PDO, and a blend of PHH and PDO in a 1:1 ratio in 1,1,1,3,3,3-hexafluoro-2-propanol (HFIP). The electrospinning system consisted of a syringe pump, a high voltage supply, and a rotating mandrel for the collection of fibers. Process parameters were optimized for the PHH solution (25% *w*/*v*) and the PHH/PDO blend (15% *w*/*v*), whereas the parameters for the PDO (15% *w*/*v*) solution were taken from the previous study [52].

For all the solutions, a positive voltage of 25 kV was applied to the syringe by the power supply and they were all delivered through a 25-gauge blunt-tip syringe needle. The PHH and PDO solutions were dispensed at a constant flow rate of 1 mL/h using a syringe pump (PHD2000, Harvard Apparatus, MA, USA), whereas the PHH/PDO blend was supplied at 1 mL/h.

For the electrospun mat with non-aligned fiber distribution, the collecting mandrel (stainless-steel rod) of 4 mm outer diameter was positioned 25 cm away from the tip of the needle and the rotation rate was 400 rpm.

For the electrospun mat with aligned fiber distribution, the aluminum collecting mandrel of outer diameter 50 mm was positioned 25 cm away from the tip of the needle and the rotation rate was 2000 rpm.

All of these scaffolds were desiccated under a vacuum for 48 h at room temperature to ensure the removal of residual solvent.

Non-porous films of PHH, PDO, and PHH/PDO blend were prepared by solution casting from HFP (15% *w*/*v*) onto siliconized Petri dishes. The solvent gets evaporated and dried films were finally desiccated under a vacuum for 48 h.

### 2.3. Structural Morphological and Chemical Characterizations

The scaffolds were characterized for physio-chemical, structural, and morphological properties. The morphological characterization of the electrospun scaffolds was carried out by imaging using a scanning electron microscope (SEM; Philips 515). The scaffolds were first sputter-coated with gold to ensure electrical conductivity. The images were analyzed with ImageJ software to determine average fiber diameter and diameter distribution.

Individual fibers of PHH, PDO, and PHH/PDO, as well as corresponding polymer films, were also examined under an atomic force microscope (AFM; TopoMetrix Explorer, Santa Clara, CA, USA) in contact mode to further investigate surface features. Images were examined with SPM-Lab software to calculate average fiber roughness and film roughness from 10 × 10 μm images.

Differential scanning calorimetry (DSC; TA Instruments Q100) was performed on the electrospun samples to determine the glass transition temperature (*T_g_*), the crystallization temperature (*T_c_*), and the melting temperature (*T_m_*), as well as their crystallinity. Percent crystallinity was determined through the ratio of enthalpy of fusion (Δ*H_f_*) of the sample to the enthalpy of fusion for 100% crystallinity ($\Delta H_{f}^{0}$):

(1) $$Crystalinity\;\left(\%\right)=\frac{\Delta H_{f}}{\Delta H_{f}^{0}}\times 100\%$$

The scaffold porosity was also calculated from the apparent density measurements. Three samples of each scaffold were cut into 5 × 5 mm squares and then weighed. Thickness was measured through a thermomechanical analysis instrument used for measuring the thermal expansion of films (TMA; TA Instruments Q400) by measuring the thickness at five various points on each scaffold sample and then averaging the thicknesses. Porosity was estimated by the following equation:

(2) $$Porosity=\left\{1-\frac{Apparent\;density\;of\;the\;scaffold}{Bulk\;density\;of\;the\;scaffold}\right\}$$

Apparent density measurement was done using the following formula [53,54]

(3) $$Apparent\;density\;of\;scaffolds=\frac{mass\;of\;the\;electrospun\;matrix}{thickness\times area}$$

The overall porosity was determined by taking the average porosity of three samples. Phosphate-Buffered Saline (PBS) uptake was also determined to evaluate the hydrophilicity of the electrospun scaffolds. Scaffold samples were initially weighed (*w*_1_) and then were immersed in phosphate-buffered saline solution (PBS; pH = 7.4) for a period of 24 h, after which they were taken out of the PBS, wiped dry with a tissue paper, and immediately weighed (*w*_2_). The percentage of PBS uptake was calculated using the following equation:

(4) $$PBS\;uptake\;\left(\%\right)=\frac{w_{2}-w_{1}}{w_{1}}$$

For chemical analysis of the samples, FTIR was performed using Thermo Nicolet nexus 4700 FT-IR spectrometers on randomly electrospun samples.

### 2.4. Biomechanical Evaluation

Electrospun specimens with randomly oriented fibers were cut into samples of 30 mm length, 2.5 mm width, and an average thickness of 165 ± 20.4 µm for dry samples, and an average thickness of 124 ± 9.3 µm for hydrated samples. Hydration was carried out by immersing the samples in PBS at room temperature for 24 h. This treatment showed no visible influence on the appearance and dimensions of the samples. Uniaxial tensile properties were measured using a BOSE Electroforce LM1 Test Bench equipped with a 22 N load cell (Bose Corporation—ElectroForce Systems Group, Eden Prairie MN, USA) at a crosshead speed of 0.1 mm/s. The ultimate tensile strength, yield strength, Young’s modulus, and elongation at break were obtained from the stress–strain curves.

## 3. Results and Discussion

### 3.1. Structural and Morphological Characterization

**Figure 1:**
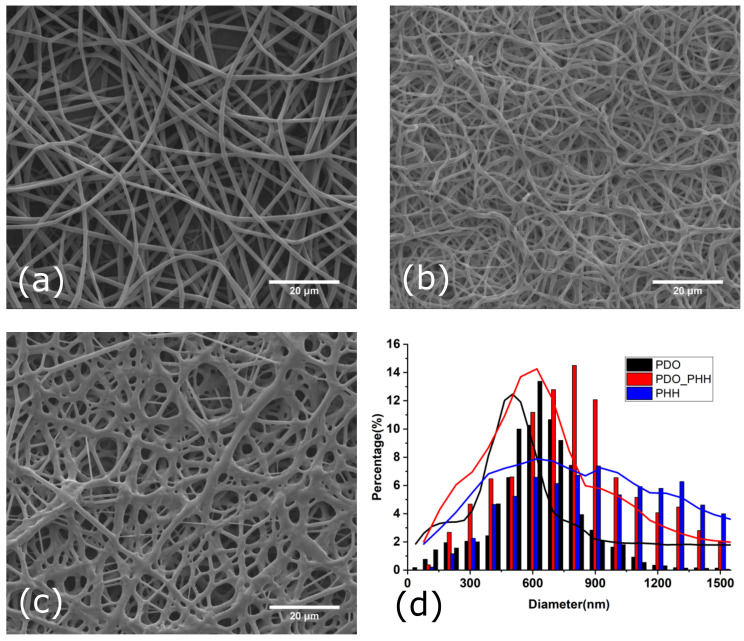
SEM images of the surface morphologies of non-aligned electrospun (**a**) PDO, (**b**) PHH/PDO, (**c**) PHH, and (**d**) diameter distribution of fibers.

The scaffolds were characterized physio-chemically and morphologically. SEM images of non-aligned electrospun PHH, PDO and the PHH/PDO blend scaffold are illustrated in Figure 1. The scaffolds prepared at the optimized electrospinning parameters show randomly oriented fiber meshes with a relatively narrow distribution of fiber diameters and without imperfections such as small polymer beads.

The electrospun PDO, PHH/PDO blend, and PHH scaffolds exhibited the diameter distribution with average values of 648 ± 157, 758 ± 297, and 1056 ± 590 nm, respectively, which is comparable to the typical diameters of collagen fibers found in natural arteries. The fiber diameters are comparable to the typical diameters of collagen fibers found in natural arteries (50 to 500 nm) [24]. Since the topology of electrospun scaffolds closely mimics that of the native extracellular matrix, these biomimetic matrices facilitate cell attachment, support cell growth, and regulate cell differentiation [2,6,25]. The SEM image of electrospun PHH reveals the physical bonding and fusing of fibers. The effect of physical bonding on the mechanical and morphological characteristics has been studied by various researchers and is reported to enhance the physical properties of the fibrous mat [55,56,57].

**Figure 2:**
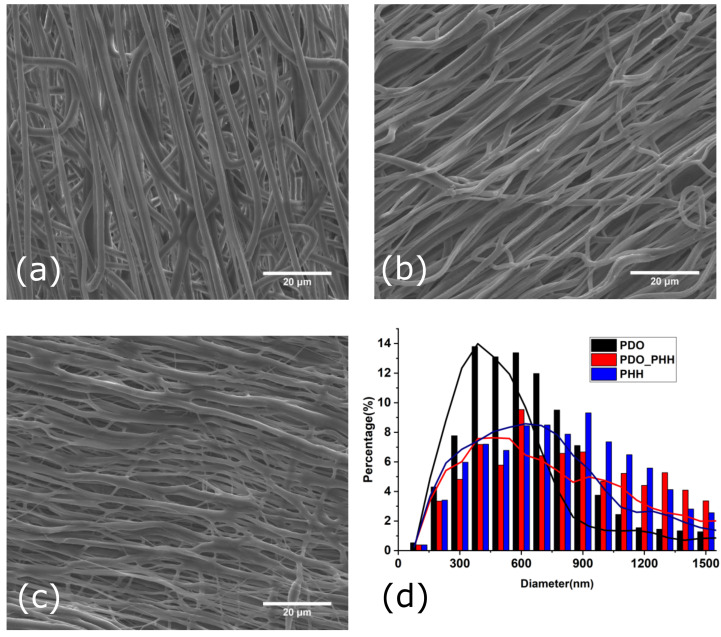
SEM images of the surface morphologies of aligned electrospun (**a**) PDO, (**b**) PHH/PDO, (**c**) PHH, and (**d**) diameter distribution of fibers.

SEM images of aligned electrospun PHH, PDO, and the PHH/PDO blend scaffold are illustrated in Figure 2.

The diameter analysis of the aligned electrospun fibers for PDO, PDO/PHH, and PHH exhibited a diameter distribution with an average value of 579 ± 255, 855 ± 600, and 900 ± 488 nm, respectively. All of these fibers are oriented in the direction of the mandrel rotation direction.

Although the stretching and recoiling of the electrospun mat were not studied in this study, the thicker diameter of the individual fibers in the mat of the electrospun PHH can be attributed to the recoil of the fibers making the diameter larger.

**Figure 3:**
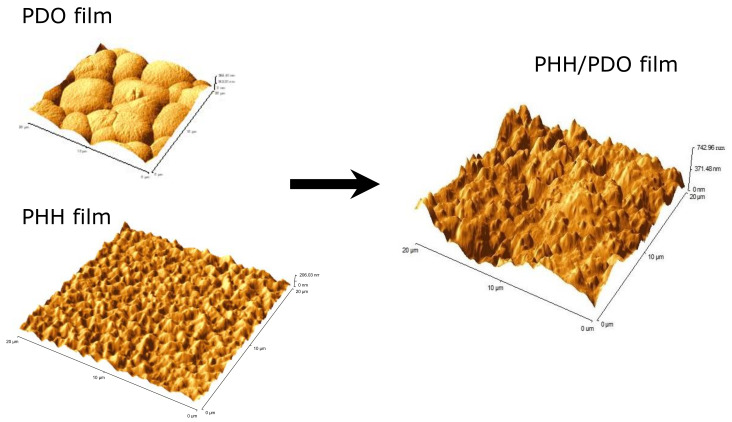
AFM image of PDO, PDO/PHH, and PHH films.

AFM (contact mode) was performed on the PHH, PDO, and PHH/PDO individual fibers, as well as on the solvent cast films of these polymer solutions. Figure 3 shows the AFM surface morphology for the films of PHH, PDO, and the PHH/PDO blend.

Amongst the film systems, PHH demonstrated the smallest roughness (RMS) of 29.70 nm, whereas PHH/PDO had the greatest of 156.32 nm. The PDO film displayed the characteristic spherulitic surface morphology, which is attributed to the high crystallinity of this polymer, and had an intermediate RMS roughness of 55.37 nm.

**Figure 4:**
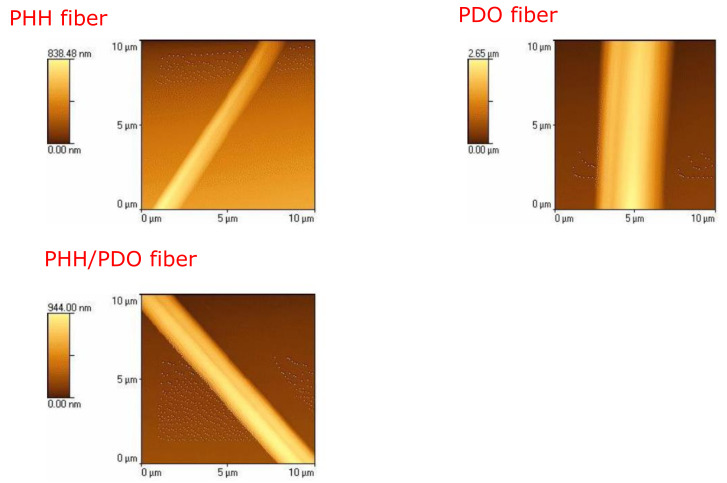
AFM image of a single fiber of PHH, PDO, and PHH/PDO films.

Figure 4 depicts the AFM images of the individual fibers. PHH fibers had a roughness (RMS) of 154.10 nm, PHH/PDO fibers had a roughness of 257.32 nm, while the PDO fibers exhibited the greatest roughness of 788.81 nm. A larger change in the roughness of the PDO fibers can be attributed to the increased surface waviness of the fibrous materials due to high crystallinity. It should be noted that a high roughness of a material can be beneficial in tissue engineering because it increases the surface area on which cells can attach to and proliferate.

**Figure 5:**
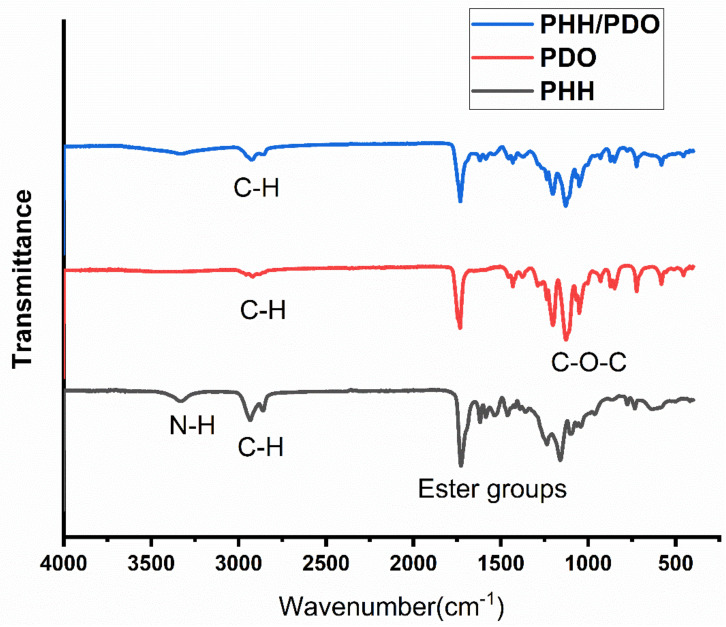
FTIR spectra of the samples.

Figure 5 represents the spectra of the electrospun PHH, PDO, and PHH/PDO blends. The peak centered around 3300 cm^−1^ in the PHH spectra represents the N-H stretching in the hydrogen-bonded urea group, which is the signature peak in the polyurethanes. The large peak at around 1700 cm^−1^ in this spectra is associated with carbonyl groups of PCL soft segments and urethane groups in PHH [35]. The characteristic C-O-C is seen in the spectra of PDO around 1125 cm^−1^ and other C-H, C=O, and O-H bands associated with the PDO are consistent with the spectra reported by Song et al. [44]. The spectrum for the blend presents peaks associated with both materials.

### 3.2. Crystallinity, Porosity, and PBS Uptake by the Electrospun Mats

**Figure 6:**
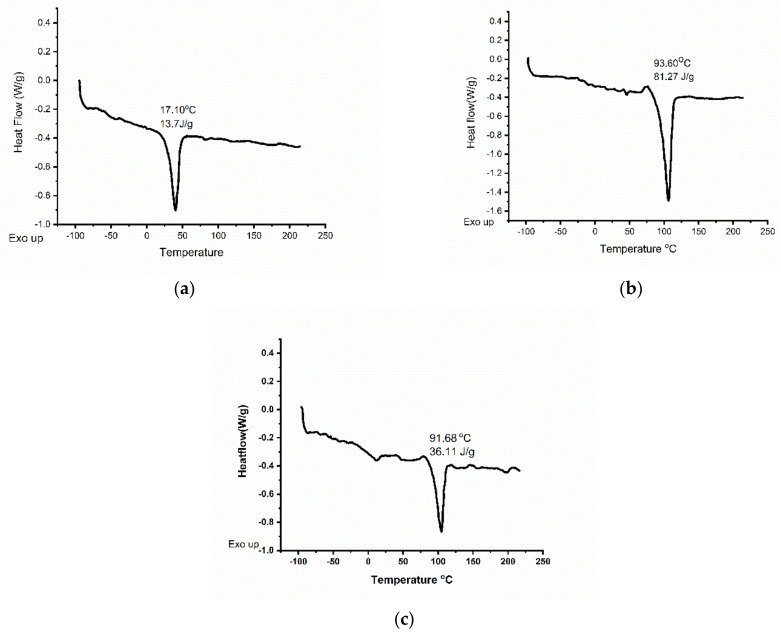
DSC images of (**a**) PHH (**b**) PDO and (**c**) PHH/PDO electrospun mats.

Comparison of crystallinity, porosity, and PBS uptake of the electrospun scaffolds is represented in Table 1. DSC experiments were performed on PHH, PDO, and blend scaffolds. The obtained results showed that PHH had a glass transition temperature, T_g_ = −60 °C, crystalline temperature, T_c_ = 4.3 °C, and melting point, T_m_ = 39.9 °C. PDO had a T_g_ = −20 °C, T_c_ = 52.4 °C, and T_m_ = 106.4 °C, and the blend had a T_g_ = −60 °C, T_c_ = −5 °C, and T_m_ = 103.4 °C. Since PHH, PDO and the PHH/PDO blend have crystalline characteristics, the heat of fusion was determined to be 13.7, 81.27, and 36.11 J g^−1^, respectively. The DSCs are depicted in Figure 6.

The presence of two Tg values for the 1:1 PHH/PDO blend in Figure 6c indicated only partial miscibility in 1:1 composition. This is in accordance with a reported miscibility study on biodegradable poly(butylene succinate) (PBS)/polydioxanone blends [58]; where 50% PDO in blend with PBS indicated semi-miscibility and more than 50% PDO indicated miscible blends. However, PDO increased to 75% in an electrospun blend with proteins degraded to show 30% weight loss within 30 days in a previous study [42]. Therefore, we opted for a 50% PDO with a 50% PHH blend system to prevent the possibility of the fast degradation in our electrospun scaffold blends.

The heat of fusion of an infinite PDO crystal, which has been determined to be 142.1 J g^−1^ [42,58], was used to calculate a crystallinity of 57.2% for the neat PDO. The degree of crystallinity of electrospun PHH, calculated taking into account the melting enthalpy for 100% crystalline PCL (148.05 J g^−1^) [59] was 11.8%. The blend exhibited a 25.6% crystallinity. The enthalpy for 100% crystallinity for the blend is calculated by averaging the enthalpy of both the constituents. The measured values of the percentage crystallinity and porosity of the blend lie in between those for individual fiber meshes.

Interestingly, the PBS uptake ability of the electrospun PHH/PDO blend scaffolds was much greater than that of the electrospun PDO-only scaffold and that of the electrospun PHH-only scaffold. The high value of the PBS absorption in electrospun PHH/PDO scaffolds is linked to a favorable combination of interconnected porosity (higher than in PHH) and decreased crystallinity due to the distribution of PHH in the PHH/PDO blend as observed by AFM. Because of the high degree of hydrophobicity and non-porous nature, the corresponding films of PHH, PDO, and PHH/PDO absorbed less than 1% PBS within 24 h.

### 3.3. Mechanical Characterization

Table 2 represents the results from the mechanical characterization of the electrospun mat and the films at dry and wet conditions.

The electrospun PDO mesh scaffolds exhibited a tensile strength (UTS) of 3.7 ± 0.5 MPa, Young’s modulus of 9.5 ± 1.0 MPa, and an elongation percentage at the break of 139.2% ± 28.1% in dry conditions. Under physiological hydrated conditions, ePDO experienced a 32.4% decrease in UTS, a 25.3% decrease in Young’s modulus, and a 7.8% increase in elongation at break. The addition of PHH to PDO changed the UTS to 2.2 ± 0.4 MPa in dry conditions and 2.0 ± 0.5 MPa in hydrated conditions, values that are intermediates between those of the two pure scaffolds. The Young’s modulus of the PHH/PDO blend was also in between the values of the pure scaffolds under both dry and hydrated conditions. The mechanical properties, in general, were found to decrease, which makes sense as water acting as the plasticizer and hence reducing the mechanical properties of polymer materials. As for the elongation at break, the blend had the lowest elongation in dry conditions and approximately the same value as the PDO scaffold under hydrated conditions. The value for elongation at break decreases in the hydrated condition for all cases except for electrospun mats with PDO, and that of the blend PHH/PDO. This is a very interesting result and could be attributed to the way crystalline PDO in fiber form absorbs water but this result needs further investigations. The mechanical properties of electrospun PHH and PHH/PDO scaffolds are comparable to those of elastomeric polymeric materials reported for cardiac tissue engineering in the literature. For example, Chen et al. have fabricated an elastomeric heart patch from polyglycerol sebacate (PGS) with modulus ranging from 0.056 to 1.2 MPa [60]. In another study, Fujimoto et al. [61] have used an elastomeric poly(ester urethane urea) (PEUU) scaffold with UTS of 0.78 MPa and a strain failure of 157% as a cardiac patch and implanted it in the sub-acute infracts developed after coronary ligation in the rat model. They have observed not only plenty of smooth muscle cells with matured contractile behavior in PEUU-implanted animals, indicating better myocardial compliance, but significantly more amounts of basic fibroblast growth factor (bFGF) and vascular endothelial growth factor (VEGF) being produced around the vessels in the PEUU patch group than in the infraction control group.

Elastomeric cardiac patches can easily recover from deformations and sustain the cyclic strain of the heart under physiological conditions due to their inherent intrinsic elasticity. However, only limited studies using nanofibrous polyurethane scaffolds have been reported as tissue engineering scaffolds so far [62,63,64]. In an in vivo rabbit study using a biodegradable poly(ester urethane) for up to 63 days, Henry et al. [62] have shown that highly porous electrospun poly(urethane) scaffolds evoked a superior host tissue and angiogenic response compared to a polyurethane film. Rockwood et al. [63] have shown that cultured cardiomyocytes in vitro on electrospun polyurethane scaffolds decreased the expression of atrial natriuretic peptide, a molecular marker that shows decreasing expression during ventricular cell maturation, than in those grown on tissue culture polystyrene. In another study, a PEUU based on a poly (ε-caprolactone) diol soft segment and a hard segment of an aliphatic diisocyanate and putrescine chain extender was found to be cytocompatible to vascular muscle cells and processed into ‘cells-micro integrated scaffolding’ by electrospraying cell-suspension concurrently with electrospinning of PEUU fibers [64]. PHH is a PCL (soft segment)-based biodegradable PEUU having HDI with a novel chain extender (AE-H-AE) (hard segment) possessing soft tissue-like mechanics [36]. Studies on protein adsorption, platelet adhesion, and thrombus formation [35,36] showed excellent blood compatibility of the PHH for potential blood-contacting applications.

## 4. Conclusions

In this study, we fabricated and tested an electrospun PHH/PDO blend (1:1) scaffold and compared the test results with those of pure PHH and PDO scaffolds. At the optimized conditions (*V* = 25 kV, *ν* = 1mL/h, *h* = 25 cm) electrospinning of 15% (*w*/*v*) PHH/PDO in HFIP solution yielded bead-free random blended micro/nanofibers with an average diameter of 758 ± 297 nm and 71.7% ± 5.3% porosity. The blended scaffold showed a low elastic modulus (~5 MPa) with a combination of the ultimate tensile strength (2 ± 0.5 MPa), and a maximum elongation (150% ± 44%) in hydrated conditions. These results are comparable to the characteristic tensile properties of cardiac or vascular tissues, and thus demonstrate the feasibility of electrospinning to fabricate a PHH/PDO blend cardiac patch that mimics the nanoscale structure and mechanical properties of native tissues. Detailed studies on the biodegradation behavior and cell–scaffold interactions of the present elastomeric scaffold should be undertaken in the future to warrant its potential use in this soft-tissue engineering.

## Figures and Tables

**Table 1:** Comparison of crystallinity, porosity, and PBS uptake by the scaffolds.

Sample	Crystallinity (%)	Porosity (%)	PBS Absorption (%)
ePHH	11.8	66.4 ± 3.8	197.1 ± 12.3
ePDO	57.2	79.2 ± 2.7	503.3 ± 34.5
ePHH/PDO	25.6	71.7 ± 5.3	852.5 ± 17.6

**Table 2:** Mechanical characterization, Comparison of tensile properties of PHH, PDO, and PHH/PDO scaffolds and films in dry and hydrated conditions.

Polymers	UTS (MPa)		Young’s Modulus (Mpa)		Elongation at Break (%)	
	Dry	Wet	Dry	Wet	Dry	Wet
PHH film	5.3 ± 0.5	4.5 ± 0.68	10.0 ± 1.5	7.8 ± 1.9	291.0 ± 44	259.0 ± 94
PDO film	5.6 ± 1.3	4.4 ± 0.89	44.4 ± 7.8	26.7 ± 2.2	28.6 ± 8.2	13.4 ± 2.3
PHH/PDO film	5.7 ± 1.1	3.2 ± 1.1	10.5 ± 1.2	23.5 ± 0.1	71.4 ± 21.5	53.6 ± 37.9
ePHH	1.1 ± 0.2	0.7 ± 0.3	1.0 ± 0.1	1.7 ± 0.7	176.3 ± 13.8	90.4 ± 19.2
ePDO	3.7 ± 0.5	2.5 ± 0.2	9.5 ± 1.0	7.1 ± 0.1	139.2 ± 28.1	150.0 ± 15.6
ePHH/PDO	2.2 ± 0.4	2.0 ± 0.5	4.8 ± 0.7	3.9 ± 1.1	73.4 ± 15.5	150.2 ± 44.4

## Data Availability

The data presented in this study are available on request from the corresponding author.

## Sample Availability

Samples of the compounds are not available from the authors.
